# The impact of attentional, linguistic, and visual features during object naming

**DOI:** 10.3389/fpsyg.2013.00927

**Published:** 2013-12-13

**Authors:** Alasdair D. F. Clarke, Moreno I. Coco, Frank Keller

**Affiliations:** ^1^Institute for Language, Cognition and Computation, School of Informatics, University of EdinburghEdinburgh, UK; ^2^Faculdade de Psicologia, Universidade de LisboaLisboa, Portugal

**Keywords:** scene perception, visual saliency, eye movements, naming, overt attention, object perception

## Abstract

Object detection and identification are fundamental to human vision, and there is mounting evidence that objects guide the allocation of visual attention. However, the role of objects in tasks involving multiple modalities is less clear. To address this question, we investigate object naming, a task in which participants have to verbally identify objects they see in photorealistic scenes. We report an eye-tracking study that investigates which features (attentional, visual, and linguistic) influence object naming. We find that the amount of visual attention directed toward an object, its position and saliency, along with linguistic factors such as word frequency, animacy, and semantic proximity, significantly influence whether the object will be named or not. We then ask how features from different modalities are combined during naming, and find significant interactions between saliency and position, saliency and linguistic features, and attention and position. We conclude that when the cognitive system performs tasks such as object naming, it uses input from one modality to constraint or enhance the processing of other modalities, rather than processing each input modality independently.

## 1. Introduction

Over the last decade, the use of natural scenes (photographs) as stimuli in vision science experiments has increased. Much of this research has concentrated on explaining the sequences of fixations and saccades made during visual tasks such as search, memorization, and free-viewing. The concept of saliency maps (Itti et al., 1998) has been an influential framework for tackling this problem and a number of different models have been proposed over the years (Toet, 2011). However, the extent to which low-level saliency can predict fixations has been questioned, and recently there has been a trend toward explaining the allocation of visual attention in terms of the objects present within the scene (Einhäuser et al., 2008b; Elazary and Itti, 2008; Nuthmann and Henderson, 2010).

**Figure 1:**
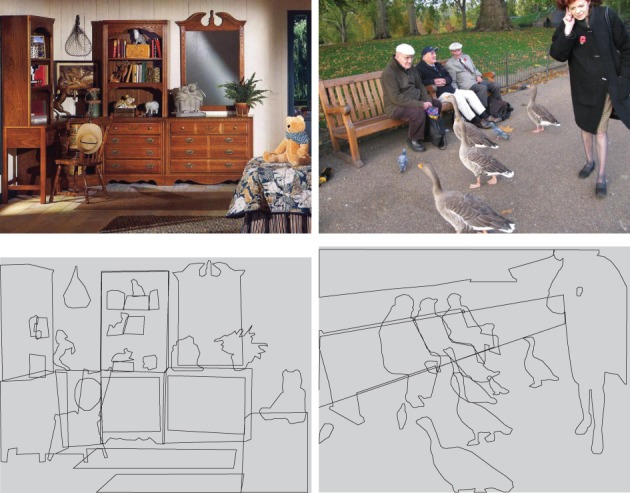
**Two example scenes with annotations**.

If the allocation of visual attention is driven by objects then this raises the question of, in a given scene, which objects are prominent and thus capture attention? For example, the two images in Figure 1 both contain many objects, some of which intuitively are more important given the context of the scene (cabinet, chair, bed and geese, bench, men, woman, respectively) than others (e.g., rug, plant and leaves, bottle, fence). Spain and Perona (2008, 2010) discuss this problem based on the concept of *object importance*, which they define as the probability of an observer mentioning the object during an object naming study. Although Spain and Perona's interests lay in machine vision, their naming task is also of interest to cognitive scientists: while eye-trackers can accurately record *where* observers look during scene-viewing, methods of self-report (free recall) such as object naming give us an insight into *what* observers perceived.

Object naming not only gives us a handle on what makes an object prominent (or important) in a given scene. It also affords us a way of investigating the role of objects in *multimodal* cognitive processing. In order to carry out a naming task, participants not only need to draw on visual features (such as position and saliency), but also on linguistic features: an object can have multiple potential names, these can vary in frequency, they can be ambiguous, and they relate semantically to other objects in the scene. By studying how attention is allocated during a multimodal task, we can therefore shed light on how the cognitive system integrates data from different modalities. It is conceivable that this integration is simply additive: when deciding which objects to name, the cognitive system independently computes prominence values in all available modalities (e.g., visual and linguistic), and then combines them into an overall prominence score. An alternative hypothesis is that the modalities influence each other, i.e., data from one modality is used to constrain or enhance the processing of other modalities. The purpose of this paper is to determine whether object naming can provide evidence for this *crossmodal guidance hypothesis*.

### 1.1. Related work

There is increasing evidence that it is objects, rather than low-level image features, that play the central role in the allocation of overt visual attention. Einhäuser et al. (2008b) carried out a series of experiments to explore the extent to which observers fixate interesting objects rather than maxima of saliency maps. Eight observers were shown a series of images which they were asked either to rate in terms of artistic interestingness or search for a specified target. After each trial they were instructed to name (by typing on a keyboard) objects that had been present in the scene. The objects named by observers were then annotated by the authors and object maps, *O*, were then constructed by setting *O*(*x*, *y*) to be the number of objects under pixel (*x*, *y*), weighted by the number of observers who named it. These proved to outperform Itti and Koch's saliency model (using the default parameters, and with no central bias) in the prediction of fixation locations: AUC = 65.1% for object maps compared to 57.8% for low-level saliency. They then proceeded to investigate object saliency scores, with the saliency of an object defined as the sum of all saliency values within its boundary, divided by the sum of the saliency scores over the whole image. They were able to show that there are strong links between object saliency and object recall, especially for objects which all of their subjects mentioned. Nuthmann and Henderson (2010) came to a similar conclusion based on an analysis of fixation locations made during a visual search experiment. They found that observers exhibited a preference for fixating the center of objects as opposed to the center of salient proto-objects (Walther and Koch, 2006). [Although, also see Leek et al. (2012) for evidence that observers show a preference for fixating areas of high curvature within objects.] The conclusion from these two studies is that low-level saliency appears to guide attention indirectly, through the objects present within a scene.

The correlation between the maxima of low-level saliency maps and objects in a scene has been explored by Elazary and Itti (2008). Using a collection of nearly 25,000 images (from the LabelMe database) they showed that the maxima of the saliency maps coincided with an annotated object in 43% of the images, considerably higher than chance (21%). The authors suggest that this is an indication that the selection of interesting objects within a scene appears to be influenced by low-level image features as well as higher-level cognitive processes. A complementary study Masciocchi et al. (2009) collected interest points from a large number of observers (over one thousand) using an online web interface and found high levels of observer agreement and significant effects of low-level saliency.

Building on these studies, Spain and Perona (2008, 2010) considered the problem of measuring the prominence of an object within a scene. They carried out an object naming study on Amazon Mechanical Turk in which observers were asked to name (by typing) ten objects that were present in the scene. The concept of object importance was then defined as the probability that a given object would be named. A set of nearly fifty features were extracted for each object, the majority of which reflected the object's position or conspicuity (Walther and Koch, 2006). Such features were then used to predict object importance using a linear regression model, achieving good performance in discriminating objects with high naming frequency from those that were rarely named.

Going beyond the low-level object properties considered by Spain and Perona, higher-level contextual properties of a scene and semantic information of individual objects are likely to have an influence on the perceived prominence of different objects within a scene. Indeed, such factors have been shown to affect fixation placement in purely visual tasks, as well as during tasks which involve the concurrent processing of visual and linguistic information. In visual search, Wang et al. (2010) found that the frequency and predictability effects on fixation duration found in the reading literature also occur in scene viewing, albeit only for small objects in a scene. This work was expanded to explore semantic guidance: observers exhibit a preference for making saccades to objects that are semantically similar to the object they are currently inspecting, and during visual search, attention is increasingly directed toward objects that are semantically similar to the target object (Hwang et al., 2011). Semantic information has been shown to influence attention within 150 ms of display onset (Gordon, 2004).

The object naming paradigm is also used by psycholinguists who study how lexical items are retrieved from memory and verbalized. Research has shown that several types of constraints, linguistic and non-linguistic, mediate the selection of lexical items and influence the associated gaze responses. On the one hand, linguistic information such as lexical frequency (Meyer et al., 1998; Almeida et al., 2007) or word length (Zelinsky and Murphy, 2000) modulates the associated gaze duration (less frequent or longer words correlate with longer gaze durations). On the other hand, the linguistic act of naming is constrained by the sentential context in which it is situated (Griffin and Bock, 1998), as well as by the semantics of surrounding objects (Damian et al., 2001; Hocking et al., 2009).

### 1.2. The present study

In the present study, we investigate the factors that influence object naming behavior. More specifically, we are interested in how information from different modalities is used by the cognitive system to determine which objects should be named. Object naming involves visual, linguistic, and attentional information; it is conceivable that these different modalities are processed independently by the cognitive system. Or, alternatively, information from one modality could guide the processing of other modalities (crossmodal guidance hypothesis).

To evaluate this hypothesis, the present study includes measures of visual attention and linguistic properties along with low-level image features such as area and saliency [unlike prior studies, e.g., (Spain and Perona, 2010)]. Furthermore, we use a fixed display time for stimulus presentation (5000 ms), and participants are instructed to only start naming objects once the stimuli has been removed from the screen. This ensures that visual attention is independent of language production (during preview), and language production is independent of visual attention (during speaking, when the scene is no longer visible). This set-up avoids biasing the naming task in favor of our hypothesis: if participants have to view a scene and speak at the same time, then visual attention and language processing are closely time-locked (e.g., Tanenhaus et al., 1995; Griffin and Bock, 2000). This time-locking is likely to artificially enhance the interaction of visual and linguistic features that we need to demonstrate as evidence for the crossmodal guidance hypothesis.

## 2. Methods

### 2.1. Stimuli

We selected 100 photographs as stimuli for this experiment. Of these, 70 were taken from the SUN09 dataset (Choi et al., 2010) and 30 from Flickr, in order to achieve a good range of scene types. Images were selected so that they contained a large number of objects, rather than being photographs focused on a single, central object. There was an approximately equal split between images that contained people and those that did not. There were also four practice trials at the start of the experiment. See Table 1 for an overview of the characteristics of the set of images used.

**Table 1:** **Overview of scene types represented**.

**People present**		**Yes**	**No**	**Scene type**	**Instances**
Inside	65	21	44	Bathroom	5
Outside	35	28	7	Bedroom	10
		49	51	Kitchen	15
				Dining room	4
				Living room	6
				Other inside	26
				Street	17
				Park	7
				Garden	3
				Other outside	7

### 2.2. Participants and procedure

Twenty-four participants were paid £6 in return for carrying out the experiment. Informed consent was obtained from each participant before the experiment started and the task was explained with written instructions. All participants were native English speakers with normal (or corrected-to-normal) vision. Participants were not screened for acuity. Before each trial was initiated, participants were required to fixate on a central fixation cross. The image was then displayed for 5000. After this period, the image was removed from display and participants were prompted with a beep to name as many objects from the scene as they could remember. They were encouraged to name at least five objects, and no specific directions were given as to what should or should not be considered an object. Participant responses were spoken; they were digitally recorded and transcribed after the experiment. Naming was self-timed and participants proceeded to the next trial by pushing a button on a response pad. The experiment lasted between 30 min and 1 h. Around half the trials from one participant were lost due to a computer error.

An Eyelink II head-mounted eye-tracker was used to monitor participants' eye-movements with a sampling rate of 500 Hz. Scenes were presented on a 21” Multiscan monitor at a resolution of 800 × 600 pixels (approximately 31°×25°, with 1° ≈ 25 pixels). Viewing was binocular although only the dominant eye was tracked. A chin rest was not used as this would have interfered with the participants' ability to produce verbal responses. Viewing distance was approximately 50 cm from the screen. Calibration was carried out at the beginning of the session and repeated again approximately halfway through the session. Some participants required more than two calibrations. Drift correction was performed between trials. The default settings for the Eyelink II fixation filter were used.

### 2.3. Objects, labels, and annotations

Rather than coming up with our own definition of what should be considered an object, we used the results from the naming experiment to generate a list of object labels for each scene. Adjectives were removed from the participants' responses (so “red car” and “white car” were both mapped to “car”) and synonyms were collapsed. Named objects that were not present in the scene were marked as mistakes, although observers were given the benefit of the doubt if there was a highly related object present in the scene. For example, in an image containing both a table and a desk, these labels were preserved as two separate categories. However, in an image that only contained a desk, any mentions of “table” were mapped onto “desk,” rather than marked as a mistake.

In general, mentions of “shirt,” “shoes,” etc. were all mapped onto “clothing” and excluded from further analysis^1^. This accounted for 0.79% of verbal responses. Similarly, references to large regions such as “sky,” “grass,” “ground,” “wall,” “floor,” and reports of the scene type were mapped to “background” (4.69% of responses).

The post-processing reduced the number of unique labels from 788 to 474. There were between 7 and 33 (mean 14.2) unique labels per image. Each images was then annotated with polygons for each instance of a named object, based on the list of object labels for the image in question. This resulted in a total of 2858 annotated polygons, with a median of 26 polygons per image. Examples are shown in Figure 1. Based on this annotation, we can now compute a mapping from the word mentions to the annotated polygons in each image. The full mapping will be released with the rest of the dataset.

### 2.4. Features

In this section, definitions of all features used in the analysis are given. Features computed from activation maps (such as attentional landscapes and saliency maps) have to be defined for categories of objects. We do this by first assigning scores to the objects within a scene by aggregating the map values (by either taking the mean or the maximum) over the pixels (*x*, *y*) that fall within the object's boundary. We then aggregate over all objects that belong to a given label, again by either taking the mean or the maximum. For example:

(1) $$\underset{{}_{O_{j}\in A}\;}{\max}\underset{(x,y)\in O_{j}}{\max}f(x,y)$$

(2) $$\underset{{}_{O_{j}\in A}}{\max}\sum_{(x,y)\in O_{j}}\frac{f(x,y)}{n_{j}}$$

for objects *O_j_* belonging to set *A* and pixels (*x*, *y*) belonging to object *O_j_*, where *n_j_* is the number of pixels belonging to *O_j_*.

#### 2.4.1. Attentional features

Mapping fixations to polygons in cluttered scenes is a non-trivial problem due to occlusion and nesting. We carried out this mapping by assigning fixations (*x*, *y*) to the polygon with the smallest area that contained it. Fixations were then mapped to *labels* by taking the union of all fixations over the polygons associated with that label.

A downside of this area-of-interest based method is that fixations which land close to, but not within, an object's boundary are not considered. A solution to this is to use attentional landscapes [also called hotspot maps, (Holmqvist et al., 2011)]. These typically involve placing Gaussian kernels over each fixation, and weighting them by fixation duration. The bandwidth of the Gaussian kernels are generally set to 1° of visual angle, to approximate foveal vision. However, these methods appear to be under-researched and therefore, we will evaluate attentional landscapes constructed using a range of bandwidths, and compare whether weighting the Gaussian kernels by fixation duration provides any benefit in the predictive power of such maps.

We will also experiment with different ways of extracting a score from attentional landscapes for given object. Each object corresponds to a binary mask, giving us a subset of pixels in an attentional landscape. We will compare using the mean, maximum, and sum of all pixels corresponding to an object. Similarly, a *mentioned label* can correspond to multiple objects within an image. (For example, a participant mentions “car” when there are three cars present in the scene.) Therefore, we will also consider defining attentional scores over *labels* as the mean, maximum or sum of the attentional scores over the objects that are represented by that label.

#### 2.4.2. Area and positional features

*Area* is perhaps the most straightforward feature: we simply take the log of the number of pixels belonging to the largest object associated with a given label. As visual attention is biased toward the center of an image (Tatler, 2007), this is likely to also have a strong influence on which objects are named. We are not aware of any previous work investigating which of the many different distance metrics give the best account for this central bias, and there are several different ways to define the distance from an object to the center of the screen. We consider two of them: *d*_*m*_ gives the distance from the center of the image to the closest point belonging to the object, while *d_c_* measures distance relative to the object's centroid. These two metrics behave slightly differently as a large object could contain the center of the image within its boundary, but still have a relatively large distance to its centroid.

#### 2.4.3. Object saliency and clutter

Features based on *saliency* are extracted in a similar way to the attentional landscape scores. We used two different saliency models: the saliency toolbox (Walther and Koch, 2006) and the low-level saliency component of the contextual guidance model (Torralba et al., 2006). Recent work by Henderson et al. (2009); Asher et al. (2013) has also shown that visual *clutter* (Rosenholtz et al., 2007) can be an effective indicator of the difficulty of finding a given target in a natural scene, therefore we included feature congestion clutter scores along with measures of visual saliency.

#### 2.4.4. Linguistic features

The *lexical frequency* of each label was obtained from the CELEX-2 database (Baayen et al., 1996). If a lexical item was not found in the database, we used Wordnet (Miller, 1995) to find the frequency of the closest synonym. In a total list of 474 unique labels, there were also 61 multi-word expressions (e.g., “dish-rack” and “cash machine”), which were not found in the database. For those cases, we took the mean frequency of its constituent words^2^.

The *semantic distance* between labels was calculated using Latent Semantic Analysis [LSA, (Landauer et al., 1998)]; as proposed by Wang et al. (2010). LSA is a widely used computational model of word meaning which measures the similarity between words based on the co-occurrence of context words within the same document. Intuitively, two words are semantically similar if they occur in similar contexts. LSA represents words as vectors of co-occurrence counts, and semantic similarity is quantified as vector distance. We built our LSA model using the British National Corpus, which contains 100 million word of text and speech (Burnard, 1995). We computed an LSA vector for each label; and the similarity between labels was measured using cosine distance.

### 2.5. Analysis

We primarily use two techniques to analyze the behavior of the features discussed above. Conditional probabilities are computed from the empirical data to show what effect individual features have on the probability of an object being named. Binomial distributions are fitted to the data to give confidence intervals. In order to assess the predictive power of different features, we use 10-fold cross validation, where a simple logistic regression model is trained on 90% of the data, and then tested in the remaining 10%; this process is repeated 10 times so that each fold functions as the test data exactly one. We compute the mean area under curve (AUC) to measure how powerful a given set of features are in predicting which objects will be named and fixated. We use a *t*-test to perform comparison between AUC of features representing the same information, e.g., distance to the center from centroid, or from closest point.

In the final part of the Results section, we investigate the crossmodal interaction of different features in predicting object naming. To achieve this, we first fit a different linear models for each family of related features (position, saliency, and linguistic features), resulting in the following set of linear equations:

(3) $$F_{p}=\beta_{p1}\log(A)+\beta_{p2}d_{m}+\beta_{p3}d_{c}$$

(4) $$F_{s}=\beta_{s1}{\text{sal}}_{T}+\beta_{s2}{\text{sal}}_{IK}+\beta_{s3}\text{clutter}$$

(5) $$F_{l}=\beta_{l1}\text{wordlength}+\beta_{l2}\text{animacy}+\beta_{l3}\text{lexfreq}+\beta_{l4}d_{s}$$

For the position model *F_p_*, the predictors used are the logarithm of the object area, log(*A*), and both measures of distance from the center: closest point, *d_m_*, and centroid, *d_c_*. For the saliency model *F_s_*, we use both measures of saliency obtained using Torralba et al. (2006), sal_*T*_, and Walther and Koch (2006), sal_*IK*_, together with visual clutter, *clutter* calculated using the Matlab toolbox developed by Rosenholtz et al. (2007). For the linguistic model *F_l_*, the predictors used are the number of characters of the label, wordlength, whether the object named is animate or inanimate, animacy, the lexical frequency of the label word in the CELEX-2 database (Baayen et al., 1996), lexfreq, and its LSA distance, *d_s_*. All predictors are normalized to range between 0 and 1.

Equations (3)–(5) each predict naming given a subset of the complete feature set. We therefore obtain a unique composite feature for each family of features (position, saliency, and linguistic) by computing the linear combination of the individual features. In particular, we multiply each predictor by its coefficient, and add the results to obtain the combined predictor [e.g., the linear combination of the position features is the expression given in Equation (3), where β_*p*1_, β_*p*2_, and β_*p*3_ are the coefficients of the predictors]. Note that we do not have an equation for attention, as we use only the best predictor observed in the analysis of attentional landscape, see section 3.1 below.

We examine crossmodal interaction, i.e., the interaction between these composite features, using linear mixed effects modeling (LME) as implemented by the R package lme4 (e.g., Baayen et al. 2008). In LME, the dependent measure is modeled as a linear function of different predictors (fixed effects), and the variance implicit in the multilevel structure of the data is accounted for based on the random variables of the design (Participants and Scenes). Since we want to infer the significant interactions directly from the data, i.e., we do not want to impose an a priori best model to the data, we perform a step-wise, forward, best-path model selection. To perform model selection, we compare nested models using a log-likelihood χ^2^-test and retain the model that returns the best statistical fit. We start with an empty model, and build its random structure first. Then, we include the fixed effects that are the experimental variables of interest (e.g., Saliency), and evaluate whether including random slopes would improve the fit. Each term (fixed or random) is included according to the impact on the log-likelihood, i.e., the term that gives the best improvement goes first. We use a conservative alpha of *p* < 0.01 as the threshold value to include or reject the term. All factors are centered to reduce collinearity. The best-path model selection procedures returns a level of Type-1 error comparable to models with maximal random effects structure (Barr et al., 2013).

Our dependent measure for the LME is a binary variable indicating whether something is mentioned (1) or not (0); thus, we use the logit link function to transform our observations to a continuous range. Therefore, the coefficients β of the model are on a logit scale, but they can be transformed back into probabilities by exponentiating the coefficients (if the reader is interested in probabilities). The predictors evaluated are the composite features given by Equations (3)–(5): Saliency, Position and Linguistics, together with best feature obtained from the attentional landscape analysis (Attention), which includes fixation duration.

In the Results section, we report and discuss the LME model coefficients of the best fitting model. The tables therefore only list those predictors that were retained in the best model. The predictors in the table are ordered in the inclusion order obtained through model selection. Moreover, as we are interested in comparing the relative importance of features, we also report and discuss standardized βs, i.e., coefficients that have been normalized so that they are all on the same scale and therefore their size can be compared directly.

In Appendix, we report a correlation matrix (Spearman's ρ) across the measures we used for all features to detect possible co-linearities, especially when we have multiple measure for the same feature (e.g., different saliency measures). This analysis shows that there are only four cases in which the correlation is higher than the level of 0.4 which is often regarded as critical for avoiding colinearity. The highest overall correlation is 0.59, between minCentroid and minPixels.

While it is important to check for colinearity in this way, we also need to emphasize that all regression models presented in this study are simple linear models in which the dependent variable (e.g., naming) predicted by a single predictor (e.g., mean semantic similarity). Moreover, the linear-mixed effect model we use to test for cross-modal interactions utilizes predictors which are linear combinations of measures within the same family of features [e.g., clutter, saliency (WK), saliency (T)]. Thus, no multiple (co-linear) predictors of the modality are concurrently present in the mixed effects model. Colinearity between predictors across modalities in the mixed model is another concern. Table 7 tabulates the relevant correlations; again, these coefficients are below the threshold of 0.4, except for two cases: Position and Attention (0.51) and Saliency and Position (0.48). This indicates that the colinearity is not a major concern in the mixed effects model.

## 3. Results

Participants made an average of 14 fixations during the 5 s display duration, and the mean number of mentioned labels per image was 5.2 (*SD* = 0.9). See Table 2 for the corresponding proportions of fixated and named objects. Overall, accuracy was high: only 3.65% of responses mistakenly referred to an object that was not present in the image. These mistakes were removed from subsequent analysis.

**Table 2:** **Mean proportions of fixated and named objects**.

	**Fixated**	**Not fixated**	**Total**
Mentioned	0.20	0.10	0.30
Not mentioned	0.20	0.50	0.70
Total	0.40	0.60	

### 3.1. Naming and attention

**Figure 2:**
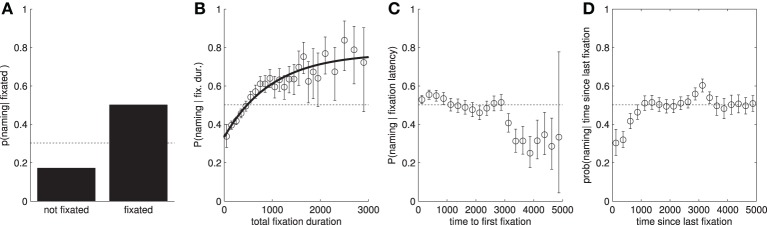
**Conditional probabilities of naming an object given attentional features**. In all cases, the dotted line shows the baseline probability of naming an object, independent of the *x*-axis. **(A)** *P*(named|fixated). We can clearly see that if a participant fixates within an object's area of interest, then they are much more likely to name the object. **(B)** *P*(named|fixated = T and total fixation duration = *x*). **(C)** *P*(named|fixated = T and time to first fixation = *x*). **(D)** *P*(named|fixated = T and time since last fixation = 5000 − *x*). Note: we have transformed the *x*-axis so the trend can easily be compared with **(C)**.

In this section we explore the extent to which the objects named by a participant can be predicted from their eye-movement behavior. We first look at whether fixated objects are more likely to be named than non-fixated objects (see Table 2 and Figure 2A. This is indeed the case. However, this is not the full story, as participants only go on to name half of objects that they fixated and a sizable proportion of non-fixated objects are named. Sustained attention on an object, in terms of the *number of fixations* and *total fixation duration* increases the likelihood that it will be named Figure 2B, but even here, *P*(named|total fixation duration = *x*) does not increase past 0.8. Both total fixation duration and the number of fixations have similar predictive power (Table 3).

**Table 3:** **AUC scores for the ability to predict which objects a participant will name based on logistic models using attentional measures**.

**Feature**	**AUC**
Number of fixations	0.706
Total fixation duration	0.706
Time to first fixation	0.544
Time since last fixation	0.535
Attentional landscape with fixation duration	0.726
Unweighted attentional landscape	0.708

We now look at whether the timings of fixations to an object can be used to help predict which objects will be mentioned. Specifically, we use the *time to initial fixation* and *time since final fixation* (time elapsed between the final fixation on an object and the offset of the image), see Figures 2C,D. Both of these measures appear to have a comparatively small effect on the conditional probability of naming a fixated object. However, we can see that objects which are not fixated within the first 3 s of the image display time are less likely to be named. Similarly, objects which are only fixated within the first second, and not re-fixated later in the trial, are also less likely to be named. Taken individually, these features score AUCs of 0.544 and 0.535, respectively (predicting which of the fixated objects will then be named). A downside of these latency-based features is that, unlike *number of fixations* and *total duration*, there is no obvious way to incorporate them in attentional landscapes which typically only include fixation location and duration information.

A weakness of the AOI analysis presented above is that we get no information for objects that are not fixated. We can see clearly from Table 2 that the lack of a fixation does not mean that an object will not be named: participants could potentially use para-foveal and peripheral vision to detect and identify objects for naming. We can extract features to represent this from attentional landscapes and give each object a score based on the density and durations of the fixations that land in its proximity.

**Figure 3:**
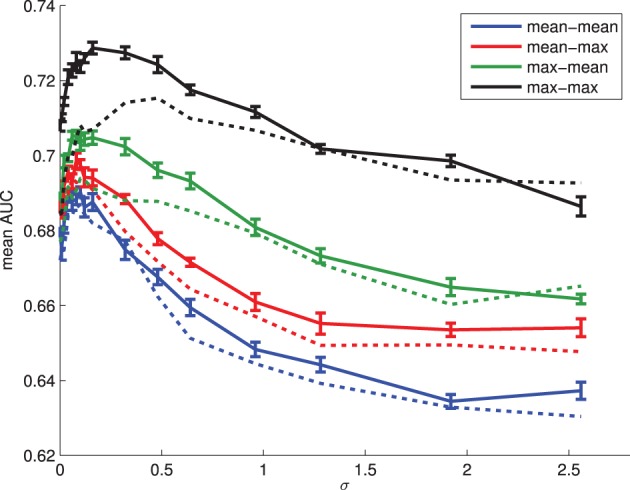
**The ability of attentional landscape scores to predict the objects an individual will name for a range of σ values (expressed in degrees of visual angle)**. Scores were obtained by first taking the maximum or mean of pixels within objects, and then aggregating over objects belonging to a class, again using the maximum or mean. In the legend above, mean-max means object scores were calculated as the maximum saliency over pixels within the object boundary, while label scores were defined as the mean over all objects associated with the object. Solid lines: attention landscape with weighting by fixation duration. Dashed lines: no weighting by fixation duration. Error bars show the standard error for the AUC scores obtained from the 10-fold cross validation.

From Figure 3 we see that the predictive power of the attentional landscape scores varies with σ, the bandwidth of the Gaussian kernel. Furthermore, attentional landscapes that weight fixations by their duration appear to perform better than, or at least as well as, those that do not. Defining attentional scores for objects as the maximum value given to the pixels within their boundary appears to perform better than using the mean, and the best performance is achieved using a relatively small kernel bandwidth, σ ≈ 0.16°. Therefore, we will use this function to provide measures of the amount of visual attention each category of object receives (max-max, weighted by fixation duration). While this method outperforms the simple AOI analyses presented in Figure 2 (equivalent to σ=0° in Figure 3), this only holds for relatively small values of σ. Using larger values, such as 1° which is more common in the literature, appears to offer no improvement on simply using *total fixation duration*.

### 3.2. Naming and object position, saliency and linguistic Factors

In the previous section we explored the role of attention on selecting objects to be named. We now consider the other features outlined in Section 2.4 and explore their role in predicting both the allocation of visual attention and their naming likelihood. In order to allow for meaningful comparisons between these features and the attentional landscape scores discussed above, we will extract features from saliency and clutter maps using the same definition as above: features for object categories (labels) are defined to be the maximum of the feature values over the associated objects in a given scene.

#### 3.2.1. Size and position

**Figure 4:**
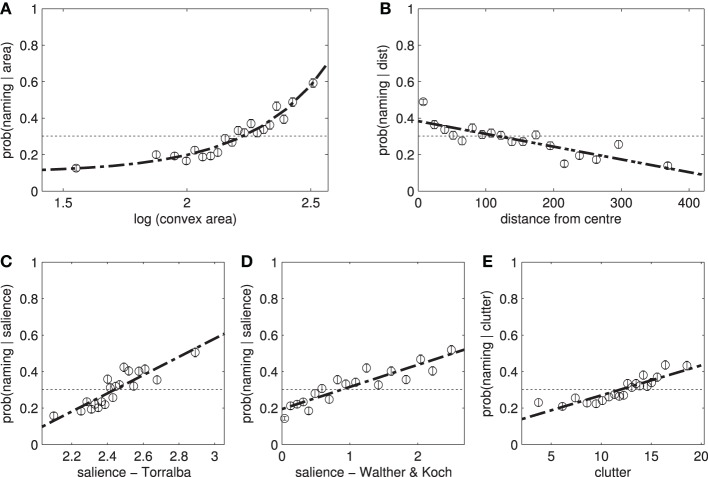
**The effect of individual features on the likelihood of naming an object**. Participants are more likely to name **(A)** large, **(B)** central items. **(C,D)** Salient items and **(E)** those with a high clutter score are also more likely to be named. These features have a similar effect on fixation locations.

We start by examining the role of area and position (Figures 4A,B). While both of these features have a considerable effect on attracting fixations and selection for naming (Table 4), the AUC scores for predicting fixation locations are greater than those predicting naming probability (according to a *t*-test on the ten AUC results from the individual models generated during tenfold cross-validation). In terms of distance metrics, measuring the distances from the closest point on the object outperforms measuring from the centroid [*t*_(18)_ = 19.86, *p* < 0.001].

**Table 4:** **AUC scores for object area and distance from center**.

**Feature**	**Fixation**	**Naming**
Log(area)	0.689	0.653
Mincentroid	0.621	0.595
MinPixels	0.691	0.622

#### 3.2.2. Saliency and clutter

The relationship between saliency and the likelihood of an object being named is shown in Figures 4C–E and the corresponding AUC scores are shown in Table 5. Saliency (WK) significantly outperforms saliency (T), *t*_(18)_ = 8.99, *p* < 0.001, and clutter, *t*_(18)_ = 14.56, *p* < 0.001, on predicting whether an object is going to be fixated, as well as, whether an object would be mentioned: *t*_(18)_ = 8.52, *p* < 0.001 and *t*_(18)_ = 19.05, *p* < 0.001, respectively. Even though the three measures of saliency are weakly correlated (ρ ≈ 0.4), refer to Table A1, they show different predictive power. This result corroborates recent work by Borji et al. (2012), which demonstrates how the predictive power saliency models changes across different experimental conditions.

**Table 5:** **AUC scores for saliency and clutter measures**.

**Feature**	**Fixation**	**Naming**
Clutter	0.610	0.585
Saliency (T)	0.626	0.615
Saliency (WK)	0.652	0.638

#### 3.2.3. Linguistic features

**Figure 5:**
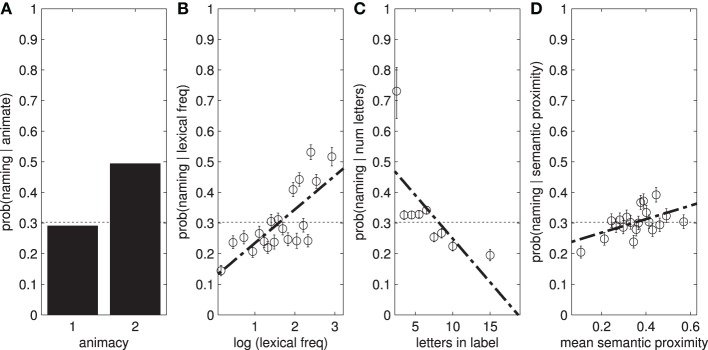
**(A)** Participants are more likely to name animate objects. The result here potentially underestimates the true effect as in some images, man, woman, and person all exist as separate labels. Some participants mention either or both of the first two labels, while some simply say “people.” **(B,C)** Show the effect of lexical frequency and word length on *P*(named|*x*), while **(D)** shows the mean semantic proximity.

Participants were far more likely to fixate and name animate objects (people) than inanimate objects (see Figure 5). There are also weak effects of word length on object naming, and a stronger effect of lexical frequency (Table 6). We also find a small effect of mean semantic proximity: participants are more likely to name objects that are semantically related to the other objects in the scene.

**Table 6:** **AUC scores for linguistic features**.

**Feature**	**Fixation**	**Naming**
Word length	0.498	0.551
Lexical frequency	0.546	0.604
Mean semantic proximity	0.514	0.530
Animacy	0.521	0.527

### 3.3. Crossmodal interaction

In our final analysis, we investigate how the four composite features proposed in this paper (Attention, Position, Saliency, and Linguistics, see section 2.5) predict object naming. We are particularly interested in interactions between these features, as they shed light on how the cognitive system integrates information from multiple modalities in a task such as object naming.

We start off by considering whether the four composite features are correlated. Table 7 presents correlation coefficients for all pairs of features. There is a substantial correlation between Position and Attention, and between Saliency and Attention. This confirms that objects that are prominent either in terms of position or in terms of saliency are attended more; furthermore, the two types of prominence (Position and Saliency) are correlated with each other. Importantly, however, there is only a weak correlation between Linguistics and any of the other features (*r* ≤ 0.18): objects that are linguistically important are not typically prominent in terms of saliency or position, or attended frequently.

**Table 7:** **Pairwise correlations (Pearson's *r*) between the composite feature used in the LME analysis**.

	**Attention**	**Position**	**Saliency**
Position	0.51		
Saliency	0.37	0.48	
Linguistics	0.15	0.17	0.18

All coefficients are statistically significant (p < 0.001)

As a next step, we fitted a mixed effects model involving the same four composite features as independent variables, and Mention (named or not) as the dependent variable. The optimal model was computed as explained in section 2.5. Table 8 reports the coefficients of those predictors that remained after model selection. We find main effects of Attention, Position, Saliency, and Linguistics. Thus, an object is more likely to be named the more it is attended to, the more prominent is in terms of its position and saliency, and the more linguistically important it is.

**Table 8:** **Coefficients for the mixed effects model analysis of Mention**.

**Predictor**	**β**	**Stand. β**	***SE***	***p***
Intercept	−0.92		0.07	0.0001
Attention	4.97	1.45	0.23	0.0001
Position	3.95	1.09	0.56	0.0001
Saliency	2.13	0.5	0.57	0.0001
Linguistics	5.22	0.92	0.93	0.0001
Attention:Position	−21.63	−0.81	1.14	0.0001
Saliency:Linguistics	23.70	0.46	2.44	0.0001
Position:Saliency	9.13	0.28	1.62	0.0001

*The normalized and centered predictors are the composite features Saliency, Position, and Linguistics, and the amount of Attention received. We give both raw coefficients (β) and standardized coefficients*.

Table 8 not only lists the raw coefficients β returned by the mixed model analysis, but also the standardized βs, i.e., coefficients that have been normalized so that they are all on the same scale and therefore their size can be compared directly. Based on the standardized βs, we find Attention to have the biggest effect on naming probability, followed by Position. Interestingly, linguistic characteristics of the object to be named are more important than their visual saliency: the standardized β of Linguistics is almost the same size as the one of Position, while the standardized β of Saliency is only about half that. As the task is inherently linguistic, objects that are linguistically important are more likely named to be than objects that are visually salient.

**Figure 6:**
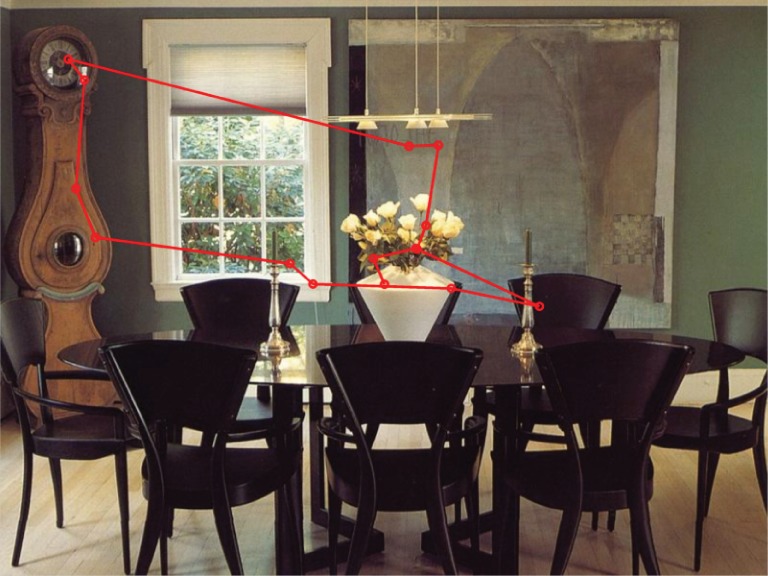
**Example illustrating the negative interaction of Attention and Position**. The objects named by the participant whose scan pattern is shown were: flowers, candle, table, chair, and clock.

Crucially, we also observe significant interactions between the composite features. A visually salient object, which is also linguistically important, is more likely to be mentioned (interaction Saliency:Linguistics). Visual saliency interacts also with Position, such that an object in a prominent position which is also more salient is more likely to be mentioned (interaction Position:Saliency). However, the likelihood of mention does not increase for all interactions: we find a negative coefficient for the interaction Attention:Position. This indicates that an object in a prominent position requires less attention in order to be named than an object in a non-prominent position. This is less counter-intuitive than it seems, as prominent objects (e.g., large and centrally located) are probably easier to detect and identify in parafoveal vision, without the needed for sustained overt attention. As an example consider the image in Figure 6, with typical scan pattern and mentioned objects. Here, the painting is in central position, and receives overt attention (several fixations), but it is not named. The clock, on the other hand, is far away from the center, receives a lot of attention, and it is named. This pattern is expected under a negative interaction of Attention and Position.

Let us return briefly to the positive interaction of Saliency and Linguistics that we observed (recall that these two composite features were not strongly correlated, see Table 7). This interaction indicates that information from these two modalities is not simply additive. Rather, linguistic information is processed in the light of saliency information: when it comes to naming, the linguistic features of more salient objects matter more than those of less salient ones (and conversely, the saliency of linguistically prominent objects has a greater effect). More generally, we can conclude that when the cognitive system performs a task such as naming, it uses input from one modality to guide (constrain or enhance) the processing of input from another modality. It does not simply process each modality separately, it integrates them interactively, presumably in the service of efficiently solving crossmodal tasks.

Note that the main effect of Saliency on naming we observe (as well as the interactions Saliency:Linguistics and Position:Saliency) challenges accounts in which visual saliency is not expected to have an impact during goal-oriented tasks (Einhäuser et al., 2008a). Naming is a goal-oriented task, and our results show that saliency (in conjunction with linguistic prominence and position) is used to determine whether an object is a viable naming candidate or not. This is unexpected if we assume that saliency is only active in free viewing an other non-goal-oriented tasks.

## 4. Discussion

In this paper we explored which factors determine whether or not an object is named in an object naming task. We found a strong link between overt attention and naming: fixated objects are more likely to be named than non-fixated objects, and a single feature based on an attentional landscape gives a good prediction of naming (AUC = 0.726). However, this is not the whole story, as one third of named objects were not fixated by observers during the 5000 ms display duration, and furthermore, only half of the objects fixated by an observer are named.

Interestingly, fixation latencies (time to onset of initial object fixations, and time elapsed since offset of final object fixation) appear to have very little to do with likelihood of naming (Figure 2). This is surprising as one would expect a primacy effect with participants fixating the important objects earlier in the scene viewing. Similarly, the objects which are viewed toward the end of the display time might be easier to remember and so we would see an effect of recency. Indeed, Irwin and Zelinsky (2002) reported such an effect in an experiment investigating visual memory for objects within a scene, with participants showing an increased ability for remembering objects that were targeted by the last three fixations of each trial. Similarly, Zelinsky and Loschky (2005) showed an increase in memory for the last three fixated objects. A potential reason for the difference with our results is that these two studies both used a paradigm in which participants were asked to report the identity of an object that had been displayed at a cued position, rather than free recall. This suggests that perhaps the participants in our study had an advantage in recalling objects fixated early and late in trial, but chose not to name them.

Beyond visual attention, we examined a range of features across different modalities and compared their ability to predict which objects were named, and which objects were fixated. We found that the positional features (an object's area and its distance from the center of the screen) have the greatest predictive power of the feature classes we have considered, and that measuring the shortest distance from an object to the center outperforms using the object's center of gravity. Although this central bias is less prominent when we are trying to explain higher-level cognitive performance (naming rather than fixating), the distance from an object to the center of the screen is still one of strongest features predicting naming.

It is possible that the effect of area is simply due to chance: if fixations were randomly distributed then larger objects would be expected to receive a higher proportion of fixations simply because they account for a greater proportion of the image's area. Taken to the extreme, a close up photograph of a single object will receive close to 100% of fixations as there is nothing else for an observer to look at. However, note that this case is not represented in our dataset: the minimum number of (annotated) objects present in a photograph was seven.

**Figure 7:**
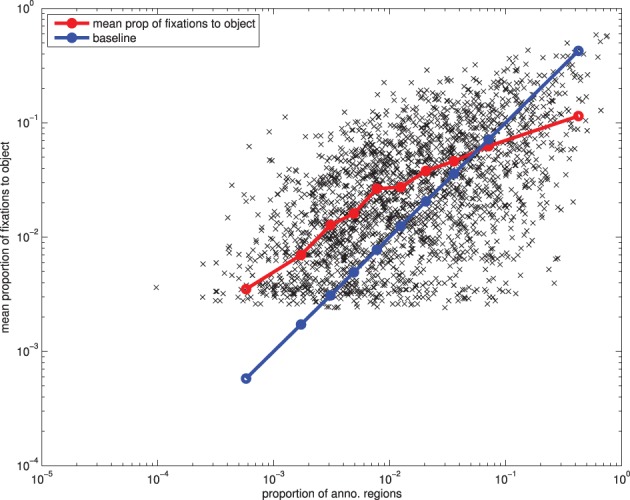
**The effect of an object's area on the proportion of fixations it receives**.

To explore the effect of area in more detail, we looked at how the proportion of fixations an object receives varies with the proportion of the image the object takes up (Figure 7). If the effect of area is simply down to chance, then the null hypothesis is for the proportion of fixations on an object to be equal to the proportion of the image taken up by the object's area. If larger objects are more salient, then we would expect the proportion of fixations to be greater than the proportion of the image. However, as can be seen in Figure 7, we actually have the opposite trend: small objects are fixated more than the null hypothesis suggests, and large objects are fixated less (although, the proportion of fixations is still positively correlated with the area of the object). This is not entirely unexpected, as a lot of the larger objects in our dataset are less interesting, background objects, such as grass, building, railing, table, fence.

There is a similar story with regards to the saliency and clutter metrics. Interestingly, we find that the saliency measure computed using the model by Walther and Koch (2006), based on the notion of proto-objects, which does not include edge information, outperforms both the saliency component of Torralba et al.'s (2006) model, as well as the clutter measure by Rosenholtz et al. (2007). As discussed also above, there is growing evidence that the efficiency of different saliency models changes as a function of the experimental conditions examined (see Borji et al. (2012) for an exhaustive analysis).

Unlike the image-based saliency and positional features, we find that linguistic factors perform better in predicting which objects are named than which will be fixated. However, we do find that linguistic factors such as lexical frequency and semantic proximity influence where we look, in line with recent work by Wang et al. (2010) and Hwang et al. (2011).

In our final analysis, we created one composite feature for each of the four features classes we considered—attention, position, saliency, and linguistic factors—and used a mixed-effects model to explore how these composite features interact. We found that each feature had a significant main effect, confirming that each modality has an impact on naming, even in a model that includes all of them (and despite the fact that the composite features are correlated). We were also able to use standardized coefficients to determine the relative importance of the four composite features, and found that Attention was the most important determinant of naming, followed by Position and Linguistics (equally important), and finally Saliency (least important).

Crucially, the mixed model also showed a number of significant interactions: Attention interacted with Position, Saliency with Linguistics, and Position with Saliency. These interactions are theoretically important, as they help us determine how the cognitive system deals with multimodal tasks such as object naming. As outlined in the Introduction, there are two competing hypotheses. One possibility is that the processing of input from multiple modalities (e.g., linguistic properties and visual properties) happens independently, in which case the effects of the various modalities should be additive (no significant interactions between factors). Alternatively the cognitive system uses input from one modality to guide (reduce or enhance) the processing of input in other modalities. In this case we should observe interactions between modalities, i.e., between the composite features that we investigated in this study. The fact that we see such interactions provides evidence for this crossmodal guidance hypothesis. For example, the interaction of Saliency with Linguistics indicates that the cognitive system, at least during object naming tasks, makes use of saliency to guide the processing of linguistic information: the linguistic prominence of a salient object is more important for naming than the linguistic prominence of a non-salient object. In other words, the processing of these modalities is not performed independently, but rather through an interactive process involving both modalities.

### Conflict of interest statement

The authors declare that the research was conducted in the absence of any commercial or financial relationships that could be construed as a potential conflict of interest.

## Footnotes

^1^Exceptions were made in cases in which several observers all named the same item of clothing.

^2^We double-checked that the mean [(*a* + *b*)/2] was an adequate approximation for compound frequency by computing also the product (*a* · *b*), as well as the minimum [min(*a*, *b*)] observed between the frequencies of its composing words. We found that the mean, minimum, and product frequencies are highly correlated (Pearson *r* = 0.95; *p* < 0.01) across all comparison.

## Appendix: correlation matrix across measures for all features

**Table A1:** **Spearman (ρ) correlation across measures of all features introduced in ourlinear regression models**.

	**Att. fix**	**Log(area)**	***d*_*C*_**	***d*_*m*_**	**sal_*T*_**	**sal_*IK*_**	**Clutter**	**Animacy**	**Lex. freq**.	**Word length**
Att. fix										
Log(Area)	0.43									
*d_C_*	−0.33	−0.06								
*d_m_*	−0.51	−0.34	0.59							
sal_*T*_	0.31	0.26	−0.21	−0.22						
sal_*IK*_	0.36	0.49	−0.19	−0.27	0.44					
Clutter	0.28	0.25	−0.14	−0.28	0.40	0.39				
Animacy	0.18	0.14	−0.14	−0.17	0.13	0.15	0.09			
Lex. freq.	0.13	0.19	−0.02	−0.06	0.16	0.16	0.14	0.36		
Word length	−0.01	−0.01	0.02	−0.02	0.01	−0.03	−0.03	−0.08	−0.31	
Semantic	0.07	0.09	−0.07	−0.07	0.07	0.09	0.13	0.06	0.29	−0.19

In Table A1, we report correlation coefficients across measures for all features discussed in this study. We observe an overall low mean correlation of 0.064 ± 0.23. This indicates that, in general, measures for different features are not co-linear. The highest value we observe is a correlation of ρ = 0.59, between minCentroid and minPixels, suggesting that most objects have regular shapes, hence the distance from centroid is already a good approximation of the position of the object.
